# The Triple-E Model: Advancing Equestrian Research with Perspectives from One Health

**DOI:** 10.3390/ani13162642

**Published:** 2023-08-16

**Authors:** Michaela M. Keener, Kimberly I. Tumlin

**Affiliations:** Sports Medicine Research Institute, University of Kentucky, Lexington, KY 40506, USA; kimberly.tumlin@uky.edu

**Keywords:** horse, horseback riding, research model, environmental factors, human–horse interaction

## Abstract

**Simple Summary:**

Sports that involve horses are complicated, and research focusing on the health and well-being of horse, rider, and environment requires collaboration across disciplines. Our team has developed a new approach called the Triple-E Model, which expands on the One Health Model. The Triple-E Model focuses on the horse (equine), rider (equestrian), and the environment. Right now, no models capture all the complexities of these interactions, even though the horse industry has a significant impact on the economy and healthcare. This paper discusses the current models and their shortcomings in equestrian sports complexities and then introduces the Triple-E Model as a solution. It allows different experts to collaborate and share their knowledge to help the equestrian community. The Triple-E Model goes beyond just looking at diseases and includes injuries and overall well-being. It is all about working together as a team to provide the best care and support for everyone involved in equestrian activities.

**Abstract:**

Equestrian sport has various welfare issues and educational needs. To address these complex interactions, we propose an integrated approach called the Triple-E Model, which focuses on the equine, equestrian, and environmental triad. A literature review of existing models suggests that complexities of these interactions are overlooked, despite the significant impact of equine industries on economics, healthcare, and animal welfare. This paper discusses current models and theories used to evaluate equine–equestrian–environmental interactions and introduces the Triple-E Model to foster multidisciplinary collaboration. Unlike the One Health triad, which focuses on disease emergence, transmission, and zoonosis, the Triple-E Model extends to non-infectious research, such as musculoskeletal injury. It promotes collaborative care and rehabilitation within the equestrian community by engaging multidisciplinary, multi-setting, and multi-sectoral teams. Given the nature of human–animal interaction and welfare considerations, this model fills the gap in understanding human–horse interactions. The paper highlights the limitations of existing models and explains how the Triple-E Model guides and encourages holistic team collaboration in the equestrian community.

## 1. Introduction

Equestrian sports are the most popular mounted sport where a human works directly with an animal through mounted activities as their teammate to complete a designated task or set of tasks in diverse environments with numerous factors contributing to the safety of all involved [1]. Unfortunately, research efforts often focus singularly on the health and safety of the horse or the human rather than looking at the equine–equestrian (horse–human) dyad simultaneously. Evaluating the relationship and interactions between the equine–equestrian dyad is critical to evaluate their impact on one another’s health and safety. Additionally, few studies have evaluated how the environment and equipment alter the interactions between the equine–equestrian dyad. To date, a literature search on equine, equestrian, and environmental research show that more research focuses on the horse than the human [2,3,4]. Although recent shifts are beginning to focus on equine–equestrian interaction, literature concentrating on both parts of the equine–equestrian dyad is sparse [5,6,7,8]. The lack of research focusing on the equine–equestrian dyad is partially due to the absence of an interdisciplinary framework for evaluating the equine–equestrian interaction and applying methods that permit cross-system assessments. Instead, assessments are discipline-specific, including veterinarian medicine, sports medicine, public health, equitation science, rehabilitation science, and engineering. A literature review evaluating current theories used in equestrianism research reported that current research tends to centralize around a single discipline, such as equitation science and Learning Theory, rehabilitation sciences and the Kinetic Chain Theory, or engineering and Dynamical Systems [9,10,11,12]. Despite the valuable contribution of this research to understanding components of equine, equestrian, and environmental topics, current theories applied in equestrian research fail to encompass all three sectors. To date, only one manuscript provides details that begin to connect equine, equestrian, and environment [13]. Stallones and colleagues provide an important explanation of the Enhanced One Welfare Framework in the Thoroughbred Industry [13]. Arguably, the Thoroughbred Industry is not representative of recreational, amateur (i.e., non-professional) competition, working equids, and therapeutic sectors of equestrianism. Previously, activity levels and perception of athleticism have shown to vary based on the level of engagement in equestrian activities [14]. Given these differences, our approach intends to guide research to create practical solutions while accounting for the complexities of the equine, equestrian, and environmental interactions for all disciplines in equestrian sport.

The absence of models addressing the complexities of the equestrian athlete triad of equine, equestrian, and environment leaves a gap in current theories. The lack of models neglects collaboration between researchers, clinicians, veterinarians, athletes, and industry stakeholders to unite ideas from the entire equestrian community. Understanding the unique complexities of the Triple-E’s (Equestrian, Equine, and Environment) is essential for addressing issues, such as rotational falls in eventing, deaths of racing Thoroughbreds, and other discipline-specific issues across the equestrian community, both as an industry and a sport. Complexities in the Triple-E’s interactions most closely align with the One Health Model.

The One Health model stems from public health and veterinary and medical care to solve complex problems impacting human, animal, and environmental health [15]. Veterinarians, and public health agents, have slowly adopted a One Health framework; however, researchers, practitioners, and others use numerous terms for the same framework, including One Health, Global Health, One Medicine, and Eco-Health [16]. Initially, One Health focused on infection and emerging diseases but has advanced into a holistic approach evaluating a multitude of issues, including climate change, food safety, and the human–animal bond [17]. However, the broadness of One Health’s reach leads to unclear boundaries and a dilution of how One Health is adopted and defined across disciplines [16]. The development of the Triple-E model is to create precise boundaries of the One Health framework applied to equestrian sports and industry. Although One Health incorporates all aspects of the Triple-E’s, the environmental factors do not include human-made environmental factors such as equipment (i.e., saddle) and course design [15,18,19,20,21]. Expanding on the One Health model to address these added complexities, we introduce the Triple-E Model. This model will help multidisciplinary teams better navigate complicated equine–equestrian–environmental problems that affect the entire equestrian community. The purpose of this theoretical paper is (1) to introduce the Triple-E Model, a framework based on the One Health model, and (2) to establish a pathway for multidisciplinary research collaboration addressing the interactions of equine–equestrian–environment (Triple-E’s).

## 2. Necessity for Expansion

Equestrianism has developed and evolved worldwide, dating back to the Bronze Age (2000–500 BC), leading to numerous disciplines within equestrian sports [22]. These disciplines include but are not limited to Thoroughbred racing, endurance riding, roping, eventing, combined driving, and vaulting [23,24]. Additionally, horses help aid varying modalities of equine-assisted activities and therapies such as therapeutic riding and behavioral interventions [25]. Though people have been analyzing equine–equestrian interaction for years, most of original explanations stemmed from word of mouth and moved around the community [26]. In the mid-20th century, researchers began collecting data and looking for answers in relation to the equine–equestrian interaction [23,27,28]. A literature review to evaluate the current theories discussed in equestrian–equine–environmental interaction research shows limitations in theories that facilitate collaboration across a multidisciplinary, multisector, and multi-setting team to evaluate variables in the three Triple-E sectors. Results of this literature review are in Table 1 and explained in the following section.

### 2.1. Current Theories in Literature

The Learning Theory (LT) helps describe the equine and equestrian process and retain information to improve their skillsets and perform together [23,26,29,30]. The LT is vital in helping understand how various training methods can heighten or lessen the natural risks of equine–equestrian interactions [31]. Like teaching humans, no single method of training will work for every horse. One of the ten training principles highlighted by the International Society of Equitation Science instructs trainers and riders to avoid a horse’s flight response, which can occur if there is an alarming stimulus in the environment [29,32,33,34,35]. The flight response is identified as a common point of injury to occur for horse and rider [30,36,37,38,39]. Unfortunately, current literature around LT does not focus on how to account or change such environmental changes in the equestrian sector.

The Open-Closed Skill Continuum Theory of motor learning and Kinetic Chain Theory of rehabilitation science with engineering roots have similar ideals [11,40]. These approaches center around how an individual is in an unstable environment or on an ever-changing surface has significantly different movement patterns, responses, muscle patterns, and skillsets when compared to an individual in a stable environment or on a stable surface [10,11,41]. Equestrian activities classify as open-skill or open-chain activities as a horse is never entirely predictable, regardless of rider’s expertise, and requires multitasking in a dynamic environment [26,40,41,42]. When applied to equestrian activities, it is common for research teams to focus on a single sector of the Triple-E Theory, including movement pattern differences of either the horse or the rider based on rider expertise, or how changing the rider’s stirrup length influences movement of the rider [5,8,43,44,45]. Although similar in nature, the literature applying the Kinetic Chain Theory in equestrian topics is sparse [46]. The next closest sport the Kinetic Chain Theory has been applied to is cycling; however, differences between a bicycle and horse are significant, making it difficult to draw true parallels [47,48]. Neither theory accounts for the environmental influence even though they both discuss differences in a dynamic environment.

Dynamical Systems Theory and Coordination Dynamics Theory provide possible insight into the equine–equestrian–environmental interactions that the previous theories do not. The Dynamical Systems Theory is a mathematical and physics-based concept, where an equation represents an initial condition with a dynamical rule specifying future conditions for the designated variables [12]. Dynamical Systems Theory is further explained in biological models through Coordination Dynamics [49]. Coordination Dynamics foundation is defined as the capability of a system to self-organize coupling across multiple subsystems, including neural, muscular, energy, biomechanics, and environment, to create harmonic movements [50]. Research analyzing the Coordination Dynamics of equine–equestrian interactions has been expanding over the past two decades. It is beginning to produce complex equine–equestrian dyad interaction models through motion capture, electromyography (EMG), and inertial measuring unit (IMU) data [5,6,44,49]. Such models of the equine–equestrian interaction, especially those of Viry and co-authors, have successfully used a coordination dynamics model to categorize the coupling and coordination dynamics of both horse and rider and their interactions during an endurance completion [6]. Unfortunately, the methods to collect the data and create coupling or predictive models are complex, leading researchers to not account for environmental factors. Viry and colleagues mentioned this lack of environmental factors as a limitation of their model [6]. They suggested environmental changes to future models, stating the environment is a subsystem in coordination dynamics, and future research should consider including the environmental subsystem in models [6,49]. Although Dynamical Systems Theory and Coordination Dynamics are beginning to dive deeper into equine–equestrian interaction, the Dynamical Systems Theory itself is intricate. The theory’s complicated roots in physics, calculus, and statistics make it daunting for disciplines outside of engineering, mathematics, and statistics. Furthermore, a theory as elaborate as the Dynamical Systems Theory makes it not easily digestible to the entire equestrian community, including trainers, riders, horse owners, and track committees.

Based on these findings, there are two critical limitations to current models. The first is the need for a simpler model for all parties included in the research agenda to follow and contribute information. Model complexity is a common problem in current research efforts in the equestrian community. The second is the need to account for environmental factors in a model, as the current models do not provide the opportunity to include these variables seamlessly. We know that equestrian environments vary by location in how equestrians engage with equine [51,52,53,54]. We postulate that environmental covariates need to be identified in future equestrian research.

### 2.2. Environmental Factors

The use of horses is worldwide, which implies that equestrian sports occur in all climates and weather conditions, impacting horse and rider experience. Some teams train in open-sided but covered arenas in southern climates all year round, while other teams ride in heated enclosed arenas half the year in northern climates [55,56]. Equestrian activities occur year-round, regardless of temperatures, storms, weather patterns, allergy cycles, humidity, and other environmental factors [24]. Understanding these environmental factors is essential because each variable can influence how both horse and rider feel separately and how they interact.

The locales in which these disciplines occur range from racing in front of thousands of cheering fans, performing for judges and silent spectators in dressage rings, following cattle through various terrains, and practicing in specialized arenas (outdoor or indoor) [23]. Additionally, each locale has different footing (surface on which the horse and rider interact), from turf tracks, to sand trails, to rubberized additives, or rocky terrain in the backcountry. These differences in surfaces and footing can change how a horse moves, thereby altering how the rider moves [57,58]. These various surfaces interact differently and cause various types of issues based on the horseshoe type, changing the horses head and sacral movement [59,60,61,62]. However, footing and locale are only a few of the vast variables in the environment that can change the Triple-E interactions.

Environmental factors do not stop at just the locale and climate difference, but also incorporate the vast difference in equipment required to perform. Changing the equipment such as the saddle, bit, and saddle pad can affect the horse’s interactions with both environment and the rider [63,64,65,66,67,68]. Different equipment facilitates communication between horse and rider, as a bit must fit the horse’s mouth properly, and depending on the style, each puts different pressure on the horse’s tongue, mouth, and face [69,70,71]. As with the locale differences, the equipment used matches the diversity of the locales and disciplines. Western saddles have more cushioning for long days in the saddle, herding livestock, and incorporates a saddle-horn in the design, used to rope livestock as needed [64]. Meanwhile, dressage and jumping saddles come from the necessity to pitch forward off the horses back while galloping and jumping obstacles during war, traveling, and hunting [22]. Finally, the Thoroughbred racing saddles are minimalistic to reduce the saddle weight and minimize influencing the horse’s freedom to move fully through the gait cycle [8,68,72]. Similarly, different bits, stirrups, stirrup lengths, and horseshoes are interchangeable and can influence the horse–rider duo interaction [43,65,67,68,69,73]. Such factors can influence the horse and rider’s movement, and an ill-fitted saddle can cause back pain to both horse and rider, making it necessary to account for these differences from a research aspect [65,67,74,75]. Understanding and adding these external variables to the environmental sector is essential as we expand the One Health model and redefine specific concepts for the Triple-E Model. Unfortunately, one of the most significant shortcomings of current theories looking at the Triple-E’s interactions fails to mention or adequately account for all three variables, with the dynamic environment being an enormous gap in these theories, especially these environmental factors.

**Table 1:** Current theories observed in a literature review of equestrian sport research and the reasons for expansion to the Triple-E Model.

Theory	Field	Basis of Theory	Current Research	Necessity for Expansion
Learning Theory	Equitation Science	Training methods can either heighten or lessen the natural risks of equine–equestrian interaction [23,26]. Specific training techniques are considerably harsher than other methods; however, no single training technique works on every horse, similar to how no single method of teaching humans works on everyone [21,27].	In equestrian activities, the main discussion is how a horse learns to reply to a fight or flight stimuli [29,32,33]. The International Society of Equitation Science implements ten training principles, one of which instructs trainers and riders to “avoid the flight response” while training horses [29]. The flight response is a standard time point linked to injury of horse and rider [29,34,35,36,37,38,39]. This response is typical when there is a change in the horse’s environment that is scary or alarming.	Learning Theory fails to account for environmental changes, even though Learning Theory highlights environmental influences as an essential factor in Learning Theory [30,31]. Learning theory helps guide trainers and riders on training practice ideals. However, it does not guide trainers or researchers on measuring and analyzing empirical evidence to help decrease risks to horses and humans.
Open-Closed Skill Continuum Theory	Motor Learning	This theory centers on how an individual responds when in an unstable environment and learns a new skill set for a given task [10,40].	When considering equestrian sports and activities, this theory automatically identifies the equine–equestrian interaction as an open-skill as a horse is never entirely predictable, regardless of rider’s expertise [26]. It is common for research teams to focus on a single sector, including movement pattern differences of either the horse or the rider based on rider expertise, or equipment changes [5,8,41,43,44].	The similarities in the foundations allow for similar shortcomings of analyzing the full scope of the Triple-E sectors. The Open-closed Skill Continuum Theory defines open-skills as dual or multitasking while in a dynamic environment [40,42,45]. Though this definition seems to fit the equine–equestrian–environmental interactions, it fails to provide structure on how to account for the environment’s dynamical influence on the complex system.
Kinetic Chain Theory (KCT)	Engineering and Rehabilitation Sciences	This theory is based on an ever-changing surface that has significantly different movement patterns, responses, muscle patterns, and skillsets when compared to an individual in a stable environment or on a stable surface [11].	Research applying the KCT to the equestrian or equine is sparse [46]. Applying the KCT research to cycling is the closest activity to equestrian activities in the current literature; however, there are apparent glaring differences between a bicycle and a horse [47,48].	Comparing an inanimate object to a live animal makes it difficult to draw comparisons between the two sports. The KCT definition suggests both the equine and equestrian are separate kinetic chains. Unfortunately, no current literature discusses how to analyze two open kinetic chains and their interactions.
Dynamical Systems Theory (DST) and Coordination Dynamics (CD)	Mathematics and Physics	In DST, an equation represents an initial condition with a dynamical rule specifying future conditions for the designated variables [12]. DST is further explained in biological models through CD [49]. CD’s foundation is defined as the capability of a system to self-organize coupling across multiple subsystems, including neural, muscular, energy, biomechanics, and environment, to create harmonic movements [50].	Research analyzing the CD of equine–equestrian interactions has been expanding over the past two decades. It is beginning to produce complex equine–equestrian dyad interaction models through motion capture, electromyography (EMG), and inertial measuring unit (IMU) data [5,6,41]. Such models of the equine–equestrian interaction, especially those of Viry and co-authors, have successfully used a CD model to categorize the coupling and coordination dynamics of both horse and rider and their interactions during an endurance competition [6].	The methods to collect the data and create predictive models are complex. Viry and colleagues omitted environmental factors due to the complexity and discussed this lack of environmental factors as a limitation of their model [6]. DST’s complicated roots in physics, calculus, and statistics make it daunting for disciplines outside of engineering, mathematics, and statistics to use. Furthermore, a theory as elaborate as DST makes it not easily digestible to the entire equestrian community, including trainers, riders, horse owners, and track committees.

## 3. The One Health Model

Incorporating the three sectors of the Triple-E’s is not a new idea; instead, it dates to Ancient Greece when Hippocrates discussed the importance of understanding the interactions of humans, animals, and the environment [76]. The One Health Theory is based on Hippocrates’s thoughts and is an interdisciplinary model supported by the World Health Organization (WHO) and Center for Disease Control and Prevention (CDC) [77,78]. The most common definition of the One Health model agreed upon between the US CDC, and the One Health Commission, is as follows: “A collaborative, multisectoral, and transdisciplinary approach (…) with the goal of achieving optimal health outcomes recognizing the interconnection between people, animals, plants, and their shared environment” [15]. To date, this approach is most commonly applicable and seen in research focusing on zoonosis, ecosystem interactions, and the spread of infectious diseases [18,20]. The One Health model is an interdisciplinary approach highlighting the importance of each sector of the human, animal, and environment models when focusing on a larger complicated outcome. Without understanding human–animal, animal–environment, and human–environment interactions, it is impossible to understand how the entire system influences an outcome and if these interactions result in a negative, positive, or neutral influence on the outcome. Without a full scope and understanding of an issue, single-discipline teams in various fields might be working for a similar cause but spending additional energy, time, and money that may or may not correctly address the overarching problem because they are unaware of variables that concern other disciplines. The purpose of the current One Health model is to facilitate collaboration across multi-disciplines to address how human, animal, and environment interactions influence a common variable of interest by providing a framework for cooperation [21,25].

Using the One Health model creates a solid foundation for expanding and specifying the definitions and uses to fit the equestrian community’s needs. Research addressing the equestrian sport and industry complexities of the equestrian, equine, and environment interactions from a multidisciplinary approach is prominent. Additionally, the Triple-E model promotes early community engagement to ensure the problem identified by researchers aligns with that of the community it directly includes. To address these unique interactions effectively, the entire equestrian community must work together. A collaborative team could include researchers, clinicians, athletes, coaches, trainers, owners, barn designers, stakeholders, and other sectors of the equestrian community. This collaborative team needs a model to guide multidisciplinary, multi-setting, multisector approaches to such problems. The purpose of the proposed Triple-E model is to do just that: guide multidisciplinary and multisector teams to evaluate the entire system when considering health, safety, guidelines, policies, and rehabilitation for the Triple-E’s.

### 3.1. Expanding One Health

**Figure 1:**
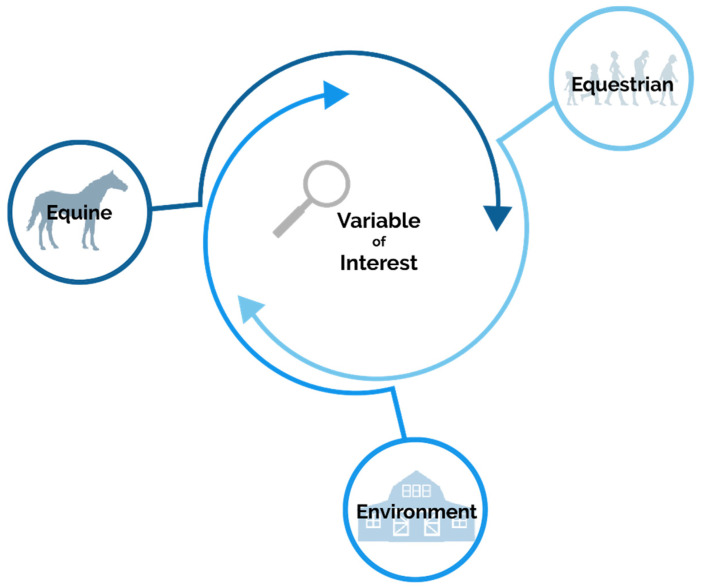
Integrating the three “E’s” into the Triple-E Model: the Equine, the Equestrian, and the Environment in which the interaction occurs.

The central tenet of the Triple-E Model (Figure 1) is based on the One Health approach. Like One Health, this is not a discipline, but rather a means to view the system from a broader perspective. Creating a holistic perspective for a research team and stakeholders provides the space and time to discuss how potential interventions, policies, and procedures can target the central research goal. A major outcome of applying this framework is to broaden the viewpoint that the central objective, such as injury prevention, is larger than the acute phase of an injury. Rather, research and evidence leading to policy changes must consider the interactions that occur outside of an acute event and appraise the interactions between all three sectors. The Triple-E Model has three outer hoops representing each “E”: equine, equestrian, and environment. Each of these hoops branches into the center to create the hoop for the central variable of interest. The branches from each hoop that create the central variable hoop have arrows to represent the feedback loop and discussion that needs to continue throughout the whole research process. The equally sized outer loops exemplify an even distribution of the Triple-E sectors’ representation regarding the central variable of interest. The central variable of interest is where the research question, sought output variables, or overarching problem to examine resides.

In Table 2, we have provided a definition and potential variables for collaborative research for each of the Triple-E sectors. These definitions are not exclusive and are adaptable to specific projects based on the multidisciplinary teams involved in a research project. Since the Triple-E model accounts for both within and between sector factors, it is necessary to collaborate with other fields, settings, and disciplines to ensure all variables are applicable and appropriate. Current factors, which are categorized into each sector of the Triple-E model, stem from previous research focusing on a single sector or interactions between two of the three sectors. Each sector should understand the current literature of the variables they discuss and suggest inclusion in the model, and how they affect the central variable of interest.

Based on the central variable of interest, the primary investigators will need to create a collaborative team across sectors, settings, and disciplines to discuss the factors which could be affecting the central variable. This team should include stakeholders from all levels of the community and sector. This process would typically start as an open forum or discussion, where all stakeholders provide insights into the variables that they think influence the central variable. Implementation and translational sciences have proven that without this community feedback loop, it is possible that the central variable is not of concern to the community it effects, or that the solution is one the community is unwilling to utilize [79,80]. The final research team may consist of a small number of investigators, but it is critical that they maintain open communication with the team they consulted prior to beginning the project. Once the research team has a list of variables from the community, the team needs to rank their importance to the central variable. This importance will depend on current literature and statistics of variables that the industry suggests as relevant, and the research team’s ability to test the variables. From here, the team needs to return to their collaborative team and discuss the options for moving forward. From there, the collaborative team can discuss the outlined variables, disciplines, settings, and sectors from each Triple-E to create a research path.

Research path options are infinite and depend on the team brought together to solve the complex issue and as always, must be based on current literature around the central variable. For example, if there is a gap in the literature on a variable ranked as extremely important, the team has the choice of two paths. One is to research in a single discipline approach to collect more information on the variable before an interdisciplinary project. The other option is to start with an interdisciplinary project. The collaborative team will have to decide on the research path. Overall, the Triple-E Model’s goal is to create a flexible path for the equestrian community to outline a research path across disciplines and settings, no matter how small or large the central variable.

### 3.2. Applying the Triple-E Model

The Triple-E Model’s flexibility allows for a multidisciplinary, multisector, multi-setting team of researchers, clinicians, barn designers, trainers, veterinarians, engineers, and other sectors of the equestrian community to have a path to guide them through the complexities of the equestrian–equine–environment interactions. As discussed above, to begin the process, the team will decide on the central variable. We will demonstrate how our model expands on Stallones and colleagues’ approach [13]. In this scenario, we are going to set the central variable to decreasing the number of injuries to Thoroughbreds and jockeys during racing sport.

The primary investigators may include a sports medicine researcher, a public health researcher, and a track veterinarian. The collaborative team will then need to include stakeholders from all sectors in discussions and discovery interviews. From the equine sector, they should include other vets, owners, and trainers. From the equestrian sector, they should talk to the riders (jockeys and exercise riders), grooms, other sports medicine researchers, the Jockey’s Guild (like a union for jockeys in the United States), and clinicians who work with the riders (athletic trainers, doctors, physical therapists, etc.). Finally, from the environmental sector, they would likely include farriers (those who trim and shod horses’ hooves), track administrators, track surface engineers, and track maintenance crews. The primary investigators will facilitate discussion with these various stakeholders, either jointly or singularly, and create a list of variables that the stakeholders believe affect the central variable. These relationships could be negative, such as increased falls related to jockeys who have more rides and longer distances across a workday [81]. They could also be positive relationships, such as decreased injury rates of Thoroughbreds on turf and synthetic tracks compared to dirt tracks [82]. Variables discussed could include data already maintained by track vets, Equibase (an online database of race information), the Jockey’s Guild, and other stakeholders that can add utility in future research. This approach could permit stakeholders to engage in deeper conversation around the central tenet; thus, recognizing the inherently complex system, which contributes to the interactions of the equine–equestrian dyad and their injury rates.

**Figure 2:**
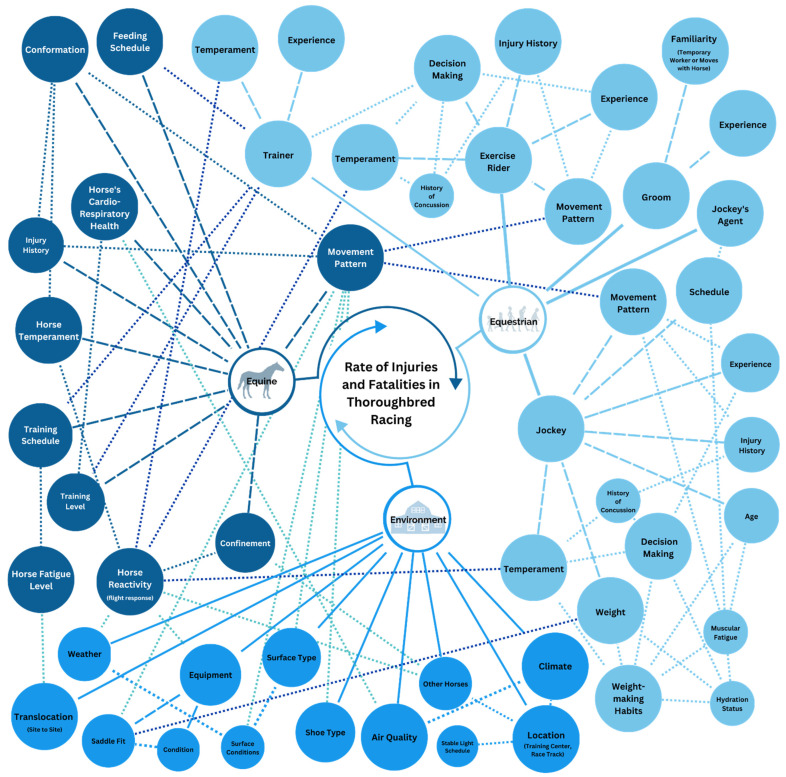
An illustration of how the Triple-E Model highlights interactions of variables in researching the rate of injury and fatalities in Thoroughbred racing from a systems map perspective.

After these discussions, the primary investigators will create a systems map (Figure 2) including the variables identified through the stakeholder feedback. The systems map can help evaluate the relationships between variables and guide the researchers where to focus [83,84,85,86]. Using the systems map in Figure 2, the researcher might decide to focus on horse movement patterns, as multiple connections from all sectors lead to this variable. The research team will then need to research the current literature on the variables connected to horse movement patterns, and their ability to evaluate each variable. From here, the primary investigators will create a research path. The proposed path of research could include single-sector research projects to provide pilot data to their idea, or a multisector study that accounts for variables in each sector. In this scenario, the research team might consider using accelerometers and saddle pressure mats to evaluate saddle fit on the horse and humans’ movement patterns. Additionally, the team might collect data on potential covariates. In this scenario, that could include surface type, surface condition, weather conditions, and the type of shoe the horse is wearing.

The primary investigators then need to return to their stakeholders and discuss their research plan. During this time, it is critical to provide adequate time and space for discussion with the various stakeholders [87]. This touch back allows the stakeholders to feel included in the process, permits stakeholders to voice comments or concerns, offers potential for community collaboration for a project, and provides an update to stakeholders. The community feedback loop included in the Triple-E model adds an important factor that Stallone’s use of a One Welfare model does not discuss but is a critical part for acceptance of a solution into a community [13,79].

As the research team works through each study, it is important to provide information back to the community of stakeholders in a way they can digest the information. Regular updates to the stakeholders allow the research team to pivot as needed and receive any new or changed information the stakeholders might have from their perspectives. Overall, the Triple-E model facilitates an interdisciplinary team to plan for future research, practice, and policy to create a safer equestrian community that focuses on all three Triple-E sectors’ health, thus expanding on current research models as described previously.

The Triple-E Model bridges the gaps between the Triple-E sectors by guiding multidisciplinary collaboration across sectors and settings to converse about the best approach to address a problem with all sectors’ interests. Overall, the Triple-E Model’s applicability to the equestrian community will improve researchers, clinicians, veterinarians, trainers, and athletes’ understanding, to form better policies and safety practices without taking away from the actual sport.

## 4. Conclusions

Current models and theories across disciplines interested in addressing the equestrian community’s problems do not facilitate a multidisciplinary, multisector, multi-setting path for researchers, owners, trainers, riders, veterinarians, and the equestrian community. The purpose of this paper was to review current theories applied to equine–equestrian–environmental interaction and introduce the newly developed Triple-E Model. This new model, expanded from the One Health model, generates a path for researchers to incorporate all equestrian community sectors into addressing problems collaboratively. The Triple-E model enables all areas of the equestrian community to create a dynamic, adaptable model incorporating equine, equestrian, and environment. Accounting for environmental factors or dynamical changes between the Triple-E’s was where other theories such as Learning Theory, Open-Closed Skill Continuum Theory, Kinetic Chain Theory, and Dynamical Systems Theory with Coordination Dynamics failed. Although these theories are appropriate for understanding different variables or interactions among the Triple-E’s within single disciplines, their lack of applicability across the triad’s complex interactions creates gaps which are filled by The Triple-E Model. The Triple-E Model provides a flexible path to guide research incorporating all equestrian community areas through multidisciplinary, multi-setting, and multisector work, incorporating equine, equestrian, and environmental factors to solve complex issues unique to the equestrian community.

## Figures and Tables

**Table 2:** Example of variables that fall within each sector of the Triple-E Model.

Triple-E Variable	Definition and List of Variables to Consider
Equestrian	Includes but is not limited to the variables of the equestrian. These variables might consist of the equestrian’s training, discipline, experience, size (height, weight, BMI, FFM/FM, leg: torso length ratio, etc.), movement pattern, health history, injury profile, asymmetries, physical fitness, PA profile, etc.
Equine	Includes but is not limited to the variables of the equine. These variables include the equine’s size, breed, movement patterns (stride frequency, length, and suspension), personality, conditioning, confirmation (neck: back length ratio), age, injury history, etc.
Environment	Includes but is not limited to environmental factors in which the horse and rider are training/working/riding/driving in, such as locale (competition, training, lesson), region/climate, season, arena type, footing type, etc. Additionally includes the equipment the duo uses, including but not limited to the saddle, bridle, saddle fit, carriage, stirrups, stirrup length, etc.

## Data Availability

Not applicable.
